# Possible mechanisms of the hypoglycaemic effect of artesunate: Gender implication

**DOI:** 10.1016/j.metop.2021.100087

**Published:** 2021-03-17

**Authors:** Abdullateef Isiaka Alagbonsi, Toyin Mohammad Salman, Sheu Oluwadare Sulaiman, Kafayat Anike Adedini, Susan Kebu

**Affiliations:** a Department of Clinical Biology (Physiology unit), School of Medicine and Pharmacy, University of Rwanda College of Medicine and Health Sciences, Huye, Rwanda; b Department of Physiology, College of Health Sciences, University of Ilorin, Ilorin, Kwara, Nigeria; c Department of Physiology, Kampala International University Western Campus, Ishaka Bushenyi, Uganda; d Department of Cell Biology, Universidade Federal de Minas Gerais, Belo Horizonte, Brazil

**Keywords:** Artesunate, Glucagon, Glucose, Glucose-6-phosphate, Insulin, Sex hormones

## Abstract

**Background:**

We investigated the mechanism of artesunate’s glucose-modulating effect especially with gender implication.

**Methods:**

Twenty-five (25) male and 25 female rats were separately and blindly allocated into five identical groups (n = 5/group). Group I (control) received 0.2 ml/kg distilled water. Groups II and III both received 2.90 mg/kg artesunate on day one, but 1.45 mg/kg from day two till day five and day fifteen respectively. Groups IV and V both received 8.70 mg/kg artesunate on day one, but 4.35 mg/kg artesunate from day two till day five and day fifteen respectively.

**Results:**

In male rats, glucose was reduced by both doses of artesunate at 5 days but increased by high dose at 15 days. Artesunate increased glycogen concentration at short duration which normalised at long duration in both genders. Artesunate increased G6P concentration only in male rats at 15 days but reduced G6Pase activity in male and female rats (except in those that received low and high doses of artesunate for 15 days). Artesunate increased insulin only in male rats treated with low dose artesunate for 5 days. Artesunate increased cortisol concentration in male but reduced it in female rats. Artesunate decreased glucagon concentration except in female rats treated with high dose for 5 days. Artesunate increased oestrogen concentration in male rats that received low dose artesunate for 5 days but reduced it in female rats that received high dose for 15 days.

**Conclusions:**

Artesunate reduces plasma glucose by reducing plasma glucagon concentrations and inhibiting liver glycogenolysis via inhibition of G6Pase activity in both sexes. Increase in insulin concentration contributed to the reduction in blood glucose caused by artesunate in male but not female rats; and artesunate-induced increase in G6P, a substrate for G6PD, could enhance NADPH generation and antioxidant enzyme activation in male rats.

## Highlights

• Reducing glucagon concentration and inhibiting G6Pase activity in both genders. .
• Increasing plasma insulin in male but not in female.
• Increasing G6P, a substrate for G6PD, in male rats.

## 1 Background

Artesunate, dihydroartemisinin, and artemether are artemisinin-derived antimalaria drugs that are very potent among various groups of drugs used in the treatment of malaria [1]. Though artemisinins can be used as a monotherapy, artemisinin combination therapies (ACTs) are preferred for the treatment of falciparum and severe malaria cases because of drug resistance and non-availability of new treatment [[2], [3], [4], [5]].

Due to the fact that artesunate is the only artemisinin-based drug with high water solubility, its intravenous administration is preferred by WHO for severe malaria treatment in both children and adults [1]. Despite the efficacy of artesunate and ACTs, the emergence of artemisinin-resistant *Plasmodium falciparum* [6] has threatened the effectiveness of ACTs. Under-dosing is a contributor to resistance to antimalarial drugs [7], especially in children [8]. Therefore, there was a proposition that increasing the dose amount and duration of artemisinins might prevent or reduce the resistance [9]. Thus, if this proposal is implemented, it might increase both positive and negative effects of artesunate. Artesunate mainly works by generating free radicals, causing alkylation of the malaria parasite’s proteins [10]. Artemisinins are generally well tolerated [11,12], and in addition to their use in the treatment of malaria, they have been shown to be useful as a therapy in many conditions such as cancer [13], immunoregulation [14,15] and glucose homeostasis.

Some adverse effects of artesunate include reduced reticulocytes [16] and post-artesunate delayed haemolysis (PADH) [17]. On glucose homeostasis, a recent report showed the hypoglycaemic and antidiabetic effects of Artemisia annua [18], the plant from which artemisinins were made [19]. In addition, the antidiabetic property of other artemisia plants has also been reviewed [20]. As an artemisinin derivative, information on the effect of artesunate on glucose homeostasis is still scanty. Artesunate and other artemisinins have been shown to cause hypoglycaemia [21], increased glucose-6-phosphate dehydrogenase activity [22], as well as the conversion of pancreatic α-cells to insulin-secreting β-like cells [23]. In addition, our recent research showed gender- and duration-based differences in blood glucose levels [24].

Despite the therapeutic uses of artesunate and the reported effects on glucose homeostasis, the mechanisms involved in its blood glucose regulation, especially in relation to gender differences, if any, have not been elucidated. Thus, this study was designed to elucidate the gender- and duration-based mechanism of the artesunate effect on glucose homeostasis by assessing some hormones and enzymes involved in blood glucose regulation.

## 2 Methods

### 2.1 Animals

Twenty-five (25) male and 25 female albino rats (170–200 g) were obtained from Bankylatt ventures Ilorin, Nigeria and acclimatised in the Central Animal House of the Faculty of Basic Medical Sciences, College of Health Sciences, University of Ilorin, Nigeria for two weeks. The animals were provided with standard diet and water *ad libitum* and maintained under standard conditions (12-hr light-dark cycle, 27–30 °C, 50–80% relative humidity). The “Principles of Laboratory Animal Care” (NIH Publication No. 85-23, revised 1985) were followed and the research was approved by our University’s Ethics Committee.

### 2.2 Experimental design

Twenty-five (25) male and 25 female rats were separately allocated by an invited neutral person who knew nothing about the study into five identical groups (n = 5/group) as follows [24]:

1. Control: Rats received 0.2 ml/kg distilled water via oral cannula.
2. Low dose artesunate for short duration: Rats were treated with artesunate at 2.90 ml/kg on day one and 1.45 mg/kg on day two till day five.
3. Low dose artesunate for long duration: Rats were treated with artesunate at 2.90 mg/kg on day one and 1.45 mg/kg on day two till day fifteen.
4. High dose artesunate for short duration: Rats were treated with 8.70 mg/kg of artesunate on day one and 4.35 mg/kg on day two till day five.
5. High dose artesunate for long duration: Rats were treated with 8.70 mg/kg of artesunate on day one and 4.35 mg/kg on day two till day fifteen.

The artesunate was freshly prepared on daily basis and administered by oral gavage with the use of cannula. The dose and duration of the drug were chosen to mimic its use in humans for low dose and short duration, while the dose and duration were tripled for high dose and long duration respectively.

### 2.3 Animal sacrifice and sample collection

At the end of the duration in each experimental group, the animals were fasted for about 18 h for the determination of fasting blood sugar and then sacrificed by decapitation under pentobarbitone anaesthesia (37 mg/kg i. p.) [25]. After sacrifice, the blood samples were collected and plasma was extracted from each sample for biochemical analysis of glucose and hormones. In addition, liver sample was collected for the analysis of glycogen, glucose-6-phosphate and glucose-6-phosphatase.

### 2.4 Biochemical analysis

#### 2.4.1 Determination of glucose and hormones

Blood glucose was estimated by glucose oxidase method using glucometer [26].

Plasma insulin, cortisol, testosterone, oestrogen (Calbiotech Inc., 1935 Cordell Ct., El Cajon, CA 92020) and glucagon (Bioassay Technology Laboratory, Yangpu Dist, Shanghai, China) were assayed spectrophotometrically (Beckman Coulter DTX 880 Multimode Detector) following the kit manufacturers’ procedure.

#### 2.4.2 Determination of liver glycogen and enzymes

The liver glycogen was determined as previously reported [27,28].

Glucose-6-phosphate (G6P) concentration was determined as previously described [29].

The glucose-6-phosphatase (G6Pase) activity was estimated using a kit from Elabscience Biotechnology Co. Ltd, Wuhan, Hubei Province, China. The ELISA kit uses Sandwich-ELISA as the method. Briefly, the micro ELISA plate provided in the kit was pre-coated with an antibody specific to Glucose-6-phosphatase. Standards or samples were added to the appropriate micro-ELISA plate wells and combined to the specific antibody. Then a biotinylated detection antibody specific for G6Pase and Avidin-Horseradish Peroxidase (Avidin-HRP) conjugate were added to each microplate well and incubated. Free components were washed away. The substrate was added to each well. Only those wells that contain G6Pase, biotinylated detection antibody and Avidin-HRP conjugate appeared blue in colour and were read spectrophotometrically at 450 nm.

### 2.5 Statistical analysis

Data were expressed as means ± standard error of mean (SEM) of the measured variables and then analysed with one-way analysis of variance, followed by a post hoc Least Significance Difference (LSD) test for multiple comparisons using Graphpad Prism 5 software (San Diego, California, USA). *p*-Values < 0.05 were taken as statistically significant.

## 3 Results

### 3.1 Artesunate reduced plasma glucose concentration

**Fig. 1:**
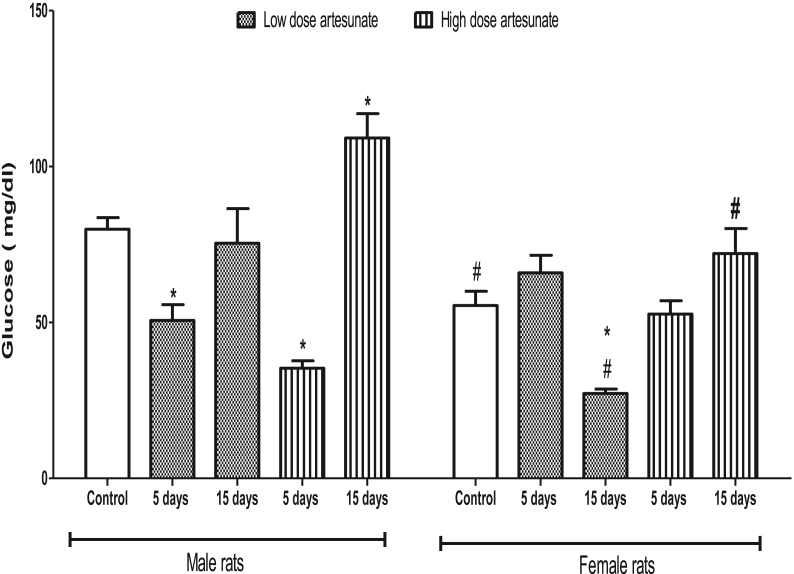
Plasma glucose concentrations in rats treated with artesunate. ∗*p* < 0.05 vs. control of the same gender, ^#^*p* < 0.05 vs. corresponding male group of the same dose and duration.

In male rats, the plasma glucose was reduced by low (50.70 ± 5.00 mg/dl) and high (35.36 ± 2.35 mg/dl) doses of artesunate at 5 days but increased by high (109.19 ± 7.80 mg/dl) and unchanged by low (75.40 ± 11.12 mg/dl) doses of artesunate at 15 days when compared to control (79.90 ± 3.77 mg/dl). In female rats, plasma glucose was reduced only by low dose artesunate at 15 days (27.23 ± 1.42 mg/dl), while other groups had no significant difference from the control (55.45 ± 4.57 mg/dl). Comparatively, female rats had lower plasma glucose than the male rats, which are especially significant in the control and groups that received both doses of artesunate for 15 days (Fig. 1).

### 3.2 Artesunate increased liver glycogen concentration at short duration which normalised at long duration

**Fig. 2:**
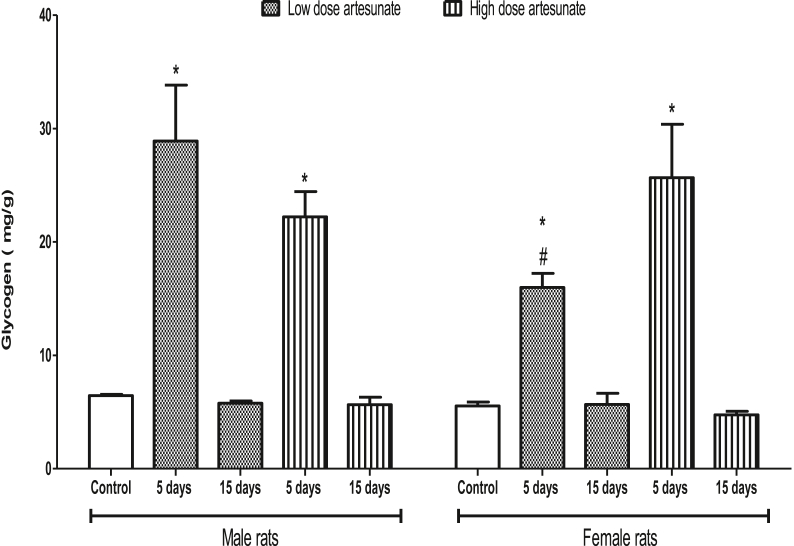
Liver glycogen concentrations in rats treated with artesunate.∗*p* < 0.05 vs. control of the same gender, ^#^*p* < 0.05 vs. corresponding male group of the same dose and duration.

In male rats, the glycogen was increased by low (28.90 ± 4.94 mg/g) and high (22.22 ± 2.23 mg/g) doses of artesunate at 5 days but unchanged by low (5.78 ± 0.21 mg/g) and high (5.65 ± 0.66 mg/g) doses of artesunate at 15 days when compared to control (6.45 ± 0.11 mg/g). Similarly, in female rats, the glycogen was increased by low (15.99 ± 1.25 mg/g) and high (25.67 ± 4.71 mg/g) doses of artesunate at 5 days but unchanged by low (5.68 ± 0.97 mg/g) and high (4.75 ± 0.32 mg/g) doses of artesunate at 15 days when compared to control (5.55 ± 0.34 mg/g). Comparatively, male and female rats that underwent the same treatment had similar glycogen concentrations, except in the female rats that received low dose artesunate for 5 days where glycogen concentration was lower than the corresponding male rats that got the same treatment (Fig. 2).

### 3.3 Artesunate increased liver G6P concentration only in male rats at 15 days

**Fig. 3:**
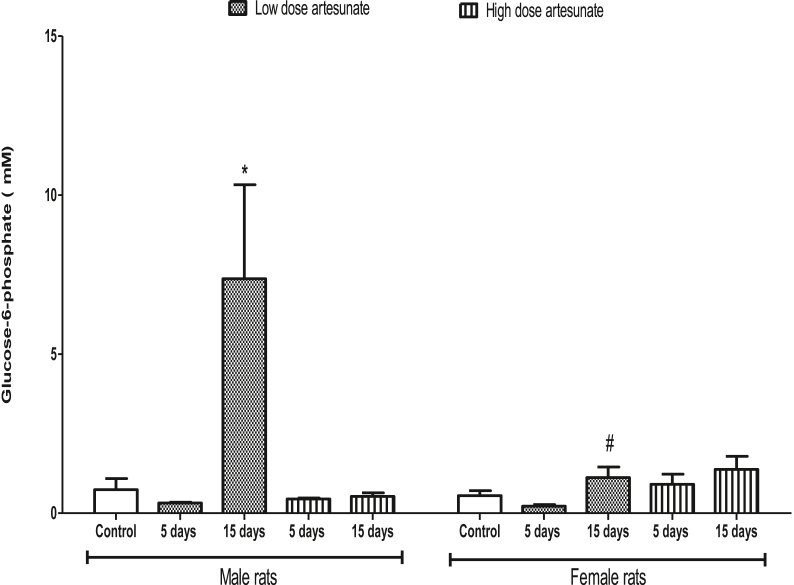
Liver glucose-6-phosphate concentrations in rats treated with artesunate. ∗*p* < 0.05 vs. control of the same gender, ^#^*p* < 0.05 vs. corresponding male group of the same dose and duration.

In male rats, there was no significant difference in the G6P concentration between treatment groups and control (0.74 ± 0.35 mM), except in those that received a low dose of artesunate for 15 days (7.37 ± 2.96 mM) where it increased. In female rats, there was no significant difference in the G6P concentration among all the treatment groups when compared to control (0.55 ± 0.16 mM). Comparatively, male and female rats that underwent the same treatment had similar G6P concentration, except in the female rats that received a low dose of artesunate for 15 days (1.12 ± 0.33 mM) where glycogen concentration was lower than the corresponding male rats that got the same treatment (7.37 ± 2.96 mM) (Fig. 3).

### 3.4 Artesunate decreased liver G6Pase concentration

**Fig. 4:**
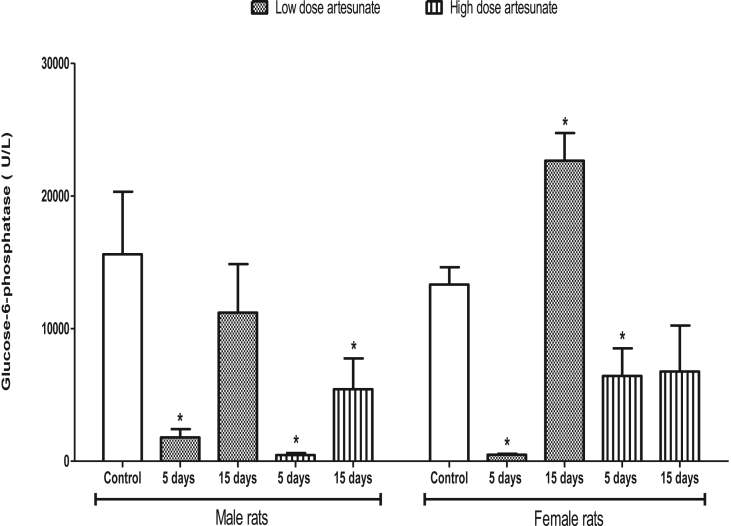
Liver glucose-6-phosphatase concentrations in rats treated with artesunate. ∗*p* < 0.05 vs. control of the same gender.

The G6Pase concentration was reduced in male rats that received low (1789.46 ± 633.73 U/L) and high (467.80 ± 143.15 U/L) doses of artesunate for 5 days, and in those that received high dose of artesunate for 15 days (5431.08 ± 2312.93 U/L) when compared to control (15599.45 ± 4717.47 U/L). The G6Pase concentration was reduced in female rats that received low (491.58 ± 63.43 U/L) and high (6428.73 ± 2081.54 U/L) doses of artesunate for 5 days, but increased and unchanged in those that received low dose (22655.85 ± 2081.25 U/L) and high dose (6759.13 ± 3476.25 U/L) artesunate respectively for 15 days when compared to control (13319.98 ± 1300.00 U/L). There was no difference in the G6Pase in the female and male rats that received similar treatment for the same duration (Fig. 4).

### 3.5 Artesunate did not affect plasma insulin concentration except in male rats treated with low dose for 5 days

**Fig. 5:**
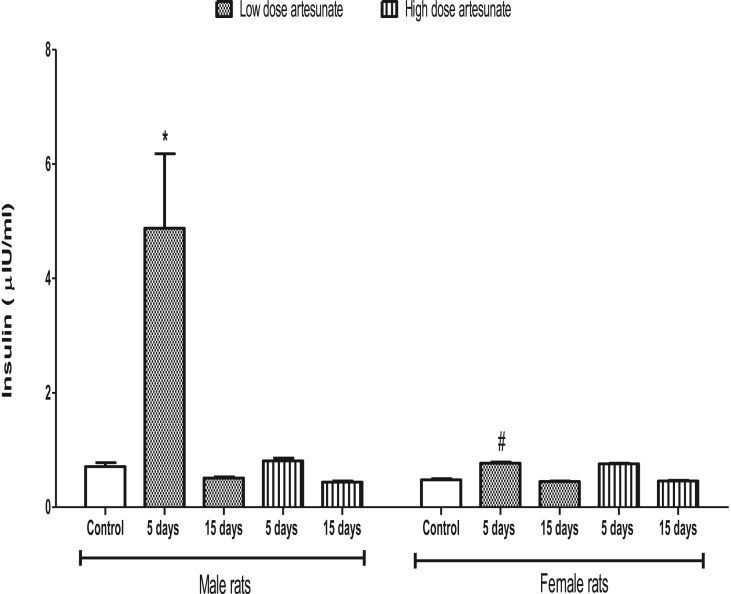
Plasma insulin concentrations in rats treated with artesunate.∗*p* < 0.05 vs. control of the same gender, ^#^*p* < 0.05 vs. corresponding male group of the same dose and duration.

Artesunate did not have any significant effect on the insulin concentration in male and female rats when compared to control, except in male rats that received low dose artesunate for 5 days (4.88 ± 1.30 μIU/ml) where insulin was increased. There were also no significant differences in the insulin levels between male and female rats that received similar treatment for the same duration, except in the male rats treated with low dose artesunate for 5 days (4.88 ± 1.30 μIU/ml) that had higher insulin than the corresponding female group with similar treatment (0.77 ± 0.02 μIU/ml) (Fig. 5).

### 3.6 Artesunate decreased plasma glucagon concentration except in female rats treated with high dose for 5 days

**Fig. 6:**
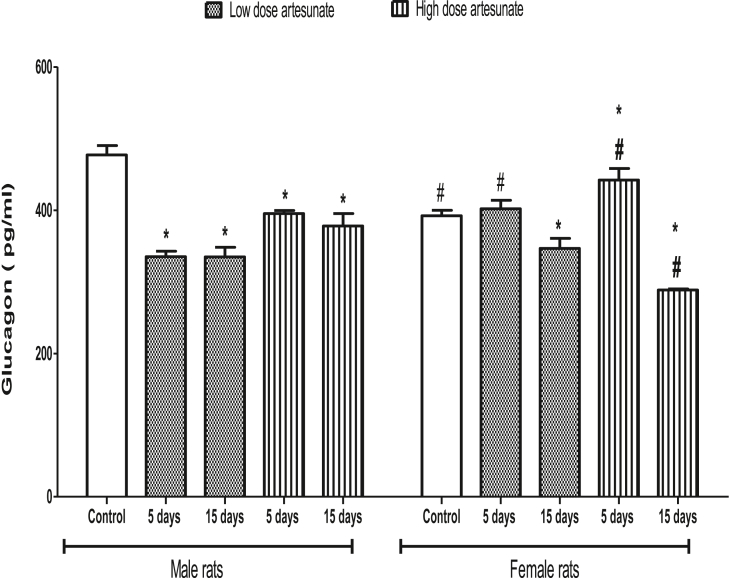
Plasma glucagon concentrations in rats treated with artesunate.∗*p* < 0.05 vs. control of the same gender, ^#^*p* < 0.05 vs. corresponding male group of the same dose and duration.

Artesunate caused significant reductions in the glucagon concentrations of all male rats’ groups when compared to control. The glucagon concentration was also reduced in female rats that received low (346.53 ± 14.17 pg/ml) and high (288.46 ± 1.61 pg/ml) doses of artesunate for 15 days but was increased and unchanged in female rats that received high (442.05 ± 16.16 pg/ml) and low (402.03 ± 11.73 pg/ml) doses of artesunate for 5 days when compared to control (392.13 ± 7.79 pg/ml). Comparatively, the glucagon concentration was higher in female rats that received low and high doses of artesunate for 5 days but lower in female rats that received high dose artesunate for 15 days when compared to their corresponding male rats with similar treatment (Fig. 6).

### 3.7 Artesunate increased plasma cortisol concentration in male but reduced it in female rats

**Fig. 7:**
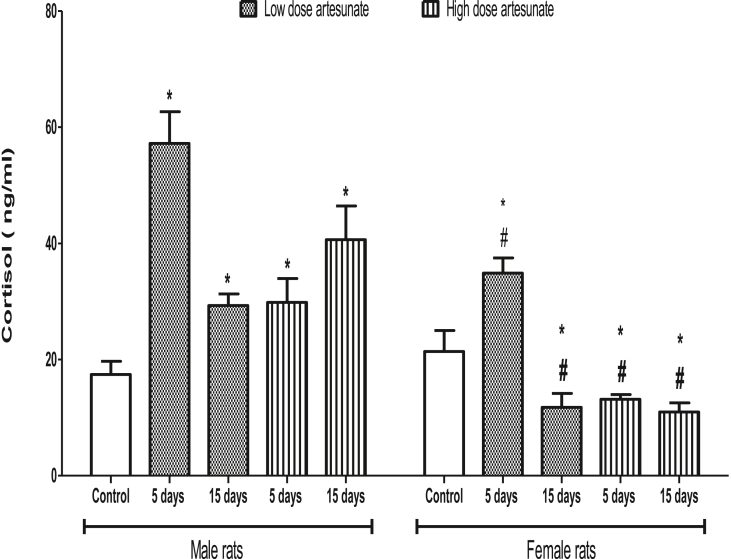
Plasma cortisol concentrations in rats treated with artesunate. ∗*p* < 0.05 vs. control of the same gender, ^#^*p* < 0.05 vs. corresponding male group of the same dose and duration.

Treatments of male rats with low dose of artesunate for 5 days (57.18 ± 5.46 ng/ml) and 15 days (29.28 ± 2.00 ng/ml), and with high dose of artesunate for 5 days (29.82 ± 4.11 ng/ml) and 15 days (40.64 ± 5.79 ng/ml) increased the cortisol concentrations when compared to control (17.42 ± 2.27 ng/ml). On the contrary, treatments of female rats with high dose of artesunate for 5 days (13.17 ± 0.79 ng/ml) and 15 days (10.96 ± 1.60 ng/ml), and with low dose of artesunate for 15 days (11.78 ± 2.37 ng/ml) decreased the cortisol concentrations when compared to control (21.40 ± 3.61 ng/ml), except in rats treated with low dose of artesunate for 5 days (34.87 ± 2.61 ng/ml) where cortisol increased. Comparatively, the female rats had lower cortisol concentrations than their corresponding male rats that received similar treatments (Fig. 7).

### 3.8 Effects of artesunate on plasma oestrogen concentration in male and female rats

**Fig. 8:**
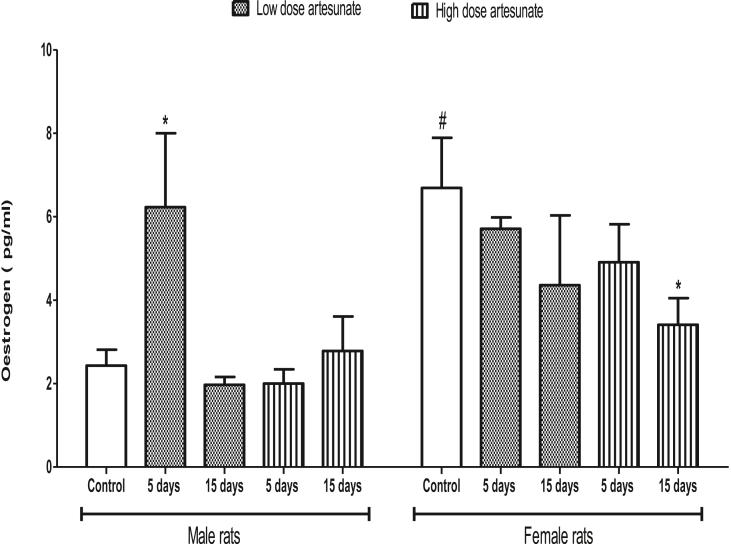
Plasma oestrogen concentrations in rats treated with artesunate.∗*p* < 0.05 vs. control of the same gender, ^#^*p* < 0.05 vs. corresponding male group of the same dose and duration.

Oestrogen concentration was not affected in male rats that received low and high doses of artesunate, except in those that received low dose for 5 days (6.23 ± 1.77 pg/ml) where it was higher than the control (2.43 ± 0.38 pg/ml). Similarly, oestrogen concentration was not affected in female rats that received low and high doses of artesunate, except in those that received high dose for 15 days (3.41 ± 0.64 pg/ml) where it was lower than the control (6.69 ± 1.20 pg/ml). Except in control rats where male had higher cortisol than the female, there was no significant difference between male and female rats treated with artesunate under similar condition (Fig. 8).

### 3.9 Effects of artesunate on plasma testosterone concentration in male and female rats

**Fig. 9:**
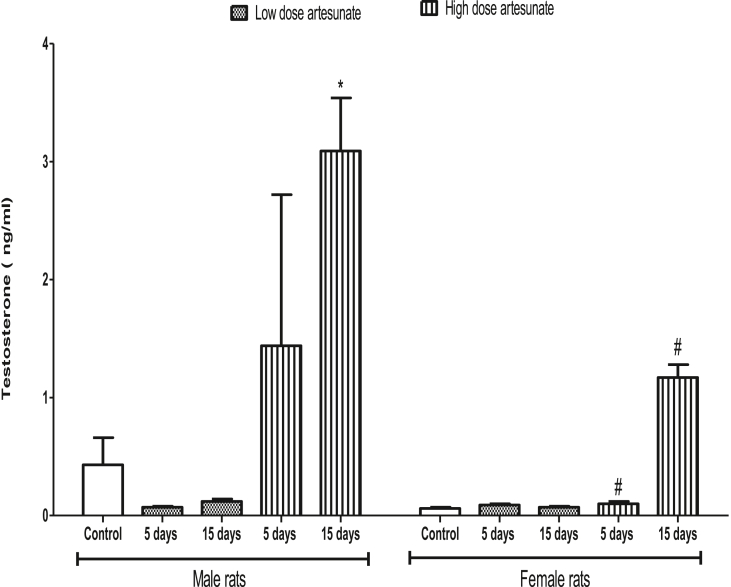
Plasma testosterone concentrations in rats treated with artesunate.∗*p* < 0.05 vs. control of the same gender, ^#^*p* < 0.05 vs. corresponding male group of the same dose and duration.

Except in male rats that received high dose of artesunate for 15 days (3.09 ± 0.45 ng/ml), artesunate did not affect testosterone concentration in all other groups when compared to control (0.43 ± 0.23 ng/ml). In female rats, artesunate did not cause any significant change in the testosterone concentration of any group when compared to control. The female rats that received high dose of artesunate for 5 days (0.10 ± 0.02 ng/ml) and 15 days (1.17 ± 0.11 ng/ml) had significantly lower testosterone than their male counterparts that received similar treatments (1.44 ± 1.28 ng/ml and 3.09 ± 0.45 ng/ml respectively) (Fig. 9).

## 4 Discussion

The reduction in blood glucose by different doses of Artesunate and other artemisinin derivatives like artemether has been reported by other researchers for a long time [21,[30], [31], [32]]. In our recent study, we also reported that artesunate produced a blood glucose-lowering effect in female rats at both 2.9 mg/kg and 8.7 mg/kg, but differential effects in the male rats - reduction with 2.9 mg/kg treatment for 5 days but increase at both 5 days and 15 days’ treatment of 8.7 mg/kg [24]. Using the same doses as in our previous study [24], we have consistently shown in this study that artesunate’s effect on the blood glucose is gender-, dose- and duration-dependent. In male rats for instance, we again observed a reduction in blood glucose by low (2.9 mg/kg) and high (8.7 mg/kg) doses of artesunate at 5 days but increase by high dose and no change by low dose of artesunate at 15 days. Our present data on female rats similarly show that blood glucose was reduced by low dose artesunate at 15 days while other groups were not affected. However, it is noteworthy that the low dose of artesunate caused hypoglycaemia on the 5th day of administration while hypoglycaemia occurred on the 15th day of administration in female rats treated with the same dose. This suggests that the onset of artesunate-induced hypoglycaemia is much earlier in male than female rats as opposed to the early occurrence of artesunate-induced haemolysis in female rats earlier reported [24]. Is the artesunate-induced reduction in blood glucose related to increase in insulin level?

The mechanism of hypoglycaemia induced by many antimalarial drugs have been suggested to be related to increase in insulin secretion. For instance, cinchona alkaloids (quinine and quinidine) increases insulin secretion by blocking ATP-sensitive potassium (K^+^ ATP) channels in the pancreatic beta cells [30,32]. Similarly, chloroquine and halofantrine increase plasma insulin and glucose uptake, leading to hypoglycaemia [21,33]. Loss of functional β-cells, as evident from the absence of serum insulin C-peptide, is a major problem of Type-1 diabetic patients. Regeneration of patient-specific insulin-producing cells have been attempted using different cell sources including hepatic cells, embryonic stem cells, exocrine cells, induced pluripotent stem cells and endocrine cells [[34], [35], [36]]. α-cells, which are developmentally closely related to β-cells, have the ability to replenish insulin-producing cells after extreme β-cell loss [37]. During development [38] and when genetically triggered in adulthood [39], overexpression of the transcription factor Pax4 converts α-cells to β-cells. Moreover, the β-cell factor Pax4 represses the α-cell master regulatory transcription factor Arx [40], the loss of which converts α-cells into β-cells [41].

Artemisinins have been identified as drugs that can confer β-cell characteristics to α-cells [23]. Artemether and its active metabolite, dihydroartemisinin, fully inhibit the Arx overexpression phenotype in Min6 cells while also inducing insulin expression in αTC1 cells. The antagonism of Arx (by inducing translocation from the nucleus to the cytoplasm and thus depleting it from the chromatin) also causes the reduction of glucagon protein levels by more than 50% [23]. The authors also convincingly showed that artemisinins induce insulin expression in the α-cells by targeting gephryin, which increases GABA signaling that will eventually suppress glucagon secretion in the α-cell. In the present study, we observed that artesunate increased insulin concentration in male rats that received low dose artesunate for 5 days, but not in other groups. Moreover, artesunate reduced glucagon concentrations of all male rats’ groups and also in female rats that received low and high doses of artesunate for 15 days. Our present study agrees with previously cited ones, and suggests that the artesunate-induced reduction in blood glucose is mediated by increase in insulin and decrease in glucagon concentrations possibly by modulating regulating proteins like Pax4 and Arx even though our present study is limited for not quantifying them.

Artesunate accumulates in the red blood cells [42] where the endoperoxidase bridge of the former is split by iron haeme present in the latter, leading to the release of ROS [43]. Studies have shown that artesunate generates free radicals, increases malondialdehyde and decreases antioxidant activities like the superoxide dismutase and catalase [24,44]. Though artesunate’s moderate pro-oxidant action beneficially serves as a mechanism for its antimalarial effect by alkylating the malaria parasite’s membrane [10], its excessive pro-oxidant action can endanger erythrocytes that host the parasite [45] and cause haemolysis [44,46]. Notwithstanding its pro-oxidant effects, artesunate has been widely reported to enhance the production and release of glucose-6-phosphate dehydrogenase (G-6-PD), a widely known second line anti-oxidant enzyme [24]. The G-6-PD is an important house-keeping enzyme that catalyses the first step of the pentose phosphate pathway, which is the sole source for the production of reducing capacity in the form of NADPH in the erythrocytes as erythrocytes lack the nucleus, the ribosomes, and the mitochondria (at maturity) that generate NADPH as in other cells [47,48]. The NADPH in turn activates glutathione reductase and catalase, which are part of the first line anti-oxidant system abundantly present in the red blood cells. The G-6-PD is the main cytoplasmic source of NADPH that prevents oxidative stress and haemolytic anaemia of the red blood cells and its deficiency leads to drug-dependent (e.g. antimalarial drug) and –independent haemolytic anaemia [45]. In fact, the overexpression of G-6-PD decreases endothelial cell oxidative stress [49] and the risk of diabetes [50,51], β-cell apoptosis and insulin resistance [52] are increased with deficiency or decrease of G6PD. The level of G-6-PD has been shown to increase in artesunate-treated erythrocytes of rats [22] and humans [53].

In our previous study, we observed that artesunate-induced increase in G-6-PD prevents haemolysis in male rats but not in female rats. We hypothesised that it is due to lower artesunate-induced lipid peroxidation in male rats compared to the female rats, which made G-6-PD’s scavenging of free radicals to sufficiently prevent haemolysis in male rats compared to the female rats with higher oxidative stress [24]. In the present study, we observed that G6P was increased only in the male rats that received low dose artesunate for 15 days, while there was no change in any of the female rats. The G6PD, a rate-limiting enzyme in the pentose phosphate pathway, acts on glucose-6-phosphate to produce 6-phosphoglucono-δ-lactone via a dehydrogenation process, during which NADPH is formed from NADP^+^. That artesunate increased glucose-6-phosphate in male rats, but not in female rats, in this study suggests that artesunate increases the substrate for G6PD that leads to accumulation of NADPH which is needed to activate other anti-oxidant enzymes (e.g. catalase and glutathione reductase) to promote free radical scavenging in the red blood cell. This further explains our previous report that artesunate prevents haemolysis in male rats but not in female rats [24].

After carbohydrate-containing meal, high postprandial insulin level stimulates glycogen synthesis by enhancing glucose entry to the liver from the blood, conversion of glucose to glucose-phosphate (G6P) with the enzyme glucokinase, and the addition of G6P molecules to the ends of the chains of glycogen. After the meal digestion and during fasting, the fall in the insulin level will stimulate glycogenolysis and gluconeogenesis that will maintain release of glucose into the blood for use by the body cells, and G6Pase is a very important enzyme in both of these metabolic processes that are paramount during fasting. In glycogenolysis, various enzyme systems remove glucose molecules from glycogen stands in the form of G6P, which remains in the cell unless it is dephosphorylated (cleaved) by the enzyme G6Pase to produce free glucose and free phosphate anion. Thus, the free glucose can be transported out of the liver to cells into the blood to maintain adequate glucose supply to many body cells. Continuous fasting-induced reduction in insulin level leads to gluconeogenesis where muscle proteins and adipose tissue triglycerides are catabolised into amino acids, free fatty acids and lactic acids. More importantly, the amino acids and lactic acid are used to form new G6P in the liver cells through the process of gluconeogenesis. Interestingly, the last step of gluconeogenesis, like that of glycogenolysis, involves the dephosphorylation of G6P by G6Pase to form free glucose and phosphate. Is artesunate-induced reduction in blood glucose related to its alternation in this key enzyme?

The glycogen storage disease type 1 (GSD 1) is an inherited disease that leads to the inability of the liver to sufficiently break down its stored glycogen and thus disrupt glucose homeostasis. It is divided into two main types (GSD 1a and GSD 1b), which differ in cause, presentation and treatment. The GSD 1a is caused by a deficiency in the enzyme G6Pase while GSD 1b is caused by a deficiency in the enzyme glucose-6-phosphate translocase (G-6-P-T) that transports G6P from the cytoplasm to the microsomes [54]. Since glycogenolysis is a major metabolic pathway by which the liver supplies glucose to the body during fasting, deficiency of both enzymes can cause severe low blood glucose and a corresponding excess glycogen storage in the liver. In our present study, we observed that the G6Pase concentration was reduced in male rats that received low and high doses of artesunate for 5 days, and in those that received high dose of artesunate for 15 days. We also observed that the G6Pase concentration was reduced in female rats that received low and high doses of artesunate for 5 days, but increased and unchanged in those that received low dose and high dose artesunate respectively for 15 days. Our present data also showed that glycogen was increased by both doses of artesunate at 5 days but unchanged by both doses at 15 days in both male and female rats. Taken together with our observation of reduction in glucagon level, the artesunate-induced reduction in G6Pase and increase in glycogen indicate that glycogenolysis is inhibited by artesunate, which led to glycogen accumulation in the liver especially during the 5 days’ duration. What led to the return of glycogen to normal level at 15 days in both male and female rats is not yet understood and needs further investigation.

The G6P has been reported to modulate the activity of 11β-hydroxysteroid dehydrogenase type 1 (11βHSD1). In GSD 1a for instance, G6P excess has been documented in the endoplasmic reticulum, which has been associated with an increase in the activity of 11βHSD1 [55]. The 11βHSD1, an endoplasmic reticulum-bound enzyme, is typically expressed in glucocorticoid receptor-rich tissues (e.g. liver, brain, lung, and adipose tissue) and is responsible for the conversion of inactive cortisone to an active cortisol [56]. The 11βHSD1 plays a key role in the development of metabolic syndrome and Cushing’s syndrome [57,58] and 11βHSD1 knockout mice are resistant to the development of metabolic syndrome [59]. In fact, a decrease in the hypothalamic-pituitary-adrenal negative feedback response has been reported in the G6PD knockout mice [60] and GSD 1a patients have reportedly shown high cortisol level [61]. Interestingly, 11βHSD1 needs NADPH (generated from the G6PD-mediated conversion of G6P to 6-phosphogluconactone) as a cofactor [62]. Though our present study did not determine the activity of 11βHSD1, we strongly speculate that the artesunate-induced increase in cortisol observed in all the male rats and in female rats that received low dose artesunate for 5 days in this study might be associated with the already-established artesunate-induced increases in G6P, G6PD and NADPH, all of which play parts in the activation of 11βHSD1. This is consistent with a previous report that accumulation of G6P in the endoplasmic reticulum fuels the G6PT-G6PD-11βHSD1 system that eventually leads to increased activation of glucocorticoids [63]. Even though the level of cortisol was increased by artesunate, it could not elicit glycogenolysis or gluconeogenesis because of artesunate-induced reduction in G6Pase.

We investigated whether some of the gender differences associated with artesunate effects reported in this study are associated with sex hormones (testosterone and oestrogen) or not. We observed that oestrogen concentration was not affected in male and female rats that received both doses of artesunate, except in male rats that received low dose for 5 days where it was increased and in female rats that received high dose for 15 days where it was reduced. We also observed that except in male rats that received high dose of artesunate for 15 days, artesunate did not affect testosterone concentration in all other male and female groups. Our no-effect observation is consistent with the previous report [64] that also observed that treatment with artesunate-amodiaquine and artemether-lumenfantrine for 3 and 6 days caused no significant effect on testosterone. The authors further showed that artesunate had no effect on the luteinising hormone, follicle stimulating hormone, and even the weights of the testis, epididymis, prostate, and seminal vesicule. It is worthy of note that the authors [64] also used 2.86 and 8.58 mg/kg of artesunate-amodiaquine for 3 days and 6 days, which are similar to our own doses, while their experimental durations are also within ours.

However, while it appears that the sex hormones played no role in the effects of artesunate generally, the sharp rise in the plasma level of oestrogen in a similar manner with insulin in the male rats treated with the low dose of artesunate for 5 days suggests that oestrogen might have contributed to the hyperinsulinaemia and hypoglycaemia observed in those rats. A previous study has reported a decrease in fasting levels of glucose upon the administration of oral oestrogen replacement in postmenopausal women [65]. In animal studies, the main steroids of the ovary, the oestrogens and the progestins, have been shown to provide a protective influence to the susceptibility to experimental diabetes [66,67]. Furthermore, an increase in basal glycaemia and an impaired glucose tolerance have been observed in ovariectomised mice as well as rats; while steroid replacement experiments indicated that a deficiency of oestrogens is mainly responsible for the deterioration of glucose tolerance [68,69]. Transdermal oestradiol replacement therapy in oestrogen-deficient postmenopausal women was shown to improve beta-cell function *in vivo* and to augment insulin secretion in response to an acute glucose challenge [70,71]. This effect was proposed to involve a tropic action of oestradiol on pancreatic islets in combination with an increase in glucose transport in the muscle and inhibition of gluconeogenesis [71]. Indeed, the islets of Langerhans have been demonstrated to express oestrogen receptors (El Seifi et al., 1981) and to show a tropic response to oestradiol treatment *in vivo* [72].

While this study is not making a strong case for the therapeutic use of artesunate as hypoglycaemic agent, it points out the fact that there is gender difference in the mechanism of action of artesunate-induced hypoglycaemia. It also suggests that dose and duration have impacts in the effect of artesunate of hormones and enzymes that modulate glucose homeostasis. However, this study is limited by our inability to measure other molecular actors in the glucose homeostasis signaling pathway apart from G6Pase. The present study suggests that artesunate causes reduction in plasma glucose by reducing plasma glucagon concentrations and inhibiting liver glycogenolysis via inhibition of G6Pase activity in both sexes. In addition, increase in plasma insulin concentration contributed to the reduction in blood glucose caused by artesunate in male but not female rats; and artesunate-induced increase in G6P, a substrate for G6PD, could enhance NADPH generation and antioxidant enzyme activation in male rats.

## Funding

This research did not receive any specific grant from funding agencies in the public, commercial, or not-for-profit sectors.

## Research involving animals

All the animals were well-catered according to the criteria outlined in the ‘Guide for the Care and Use of Laboratory Animals’ prepared by the National Academy of Science and approved by the Ethical Research Committee of the University of Ilorin, Nigeria.

## Consent for publication

Not applicable.

## Authors’ contributions

TMS conceived, designed and supervised the study. AK and AA carried out the study. IAA and SOS analysed and interpreted the data, and drafted the manuscript. All authors read and approved the final manuscript to be published.

## Declaration of competing interest

The authors declare that they have no competing interest.
